# Proteomics reveals molecular subtypes in traumatic brain injury patients: new perspectives for precision treatment strategies

**DOI:** 10.3389/fimmu.2025.1606268

**Published:** 2025-05-08

**Authors:** Suqin Zhang, Qi Xu

**Affiliations:** ^1^ Department of Neurosurgery, Sinopharm Tongmei General Hospital, Datong, China; ^2^ Department of Gastroenterology, Second Affiliated Hospital of Dalian Medical University, Dalian, China

**Keywords:** proteomics, molecular subtypes, traumatic brain injury, precision treatment strategies, biomarker discovery

## Introduction

Traumatic Brain Injury (TBI) is a leading neurological condition worldwide, with high rates of disability and mortality. The rising incidence of TBI, along with its long-term neurological effects, poses a significant challenge to healthcare systems (1). Although there have been advances in clinical diagnostics and therapeutic strategies in recent years, early and accurate diagnosis, personalized prognostic assessments, and targeted treatments for TBI continue to face obstacles.

Current clinical practices mainly rely on imaging techniques such as CT and MRI, as well as traditional biomarkers like GFAP and NFL, to differentiate TBI patients from healthy individuals and those with non-TBI trauma (2–5). However, these diagnostic approaches often focus on single indicators or specific phenotypic traits, which hampers efforts to systematically understand the complex pathophysiological mechanisms underlying TBI heterogeneity. This includes aspects like neuroinflammatory cascades, the dynamic progression of axonal injury, and disruptions in metabolic-autophagy pathways.

In light of these challenges, proteomic technologies present a promising avenue. By enabling high-throughput analysis of protein expression fluctuations in body fluids such as plasma and cerebrospinal fluid, these technologies provide valuable insights into the molecular mechanisms of TBI (6, 7).

## Result

**Figure 1:**
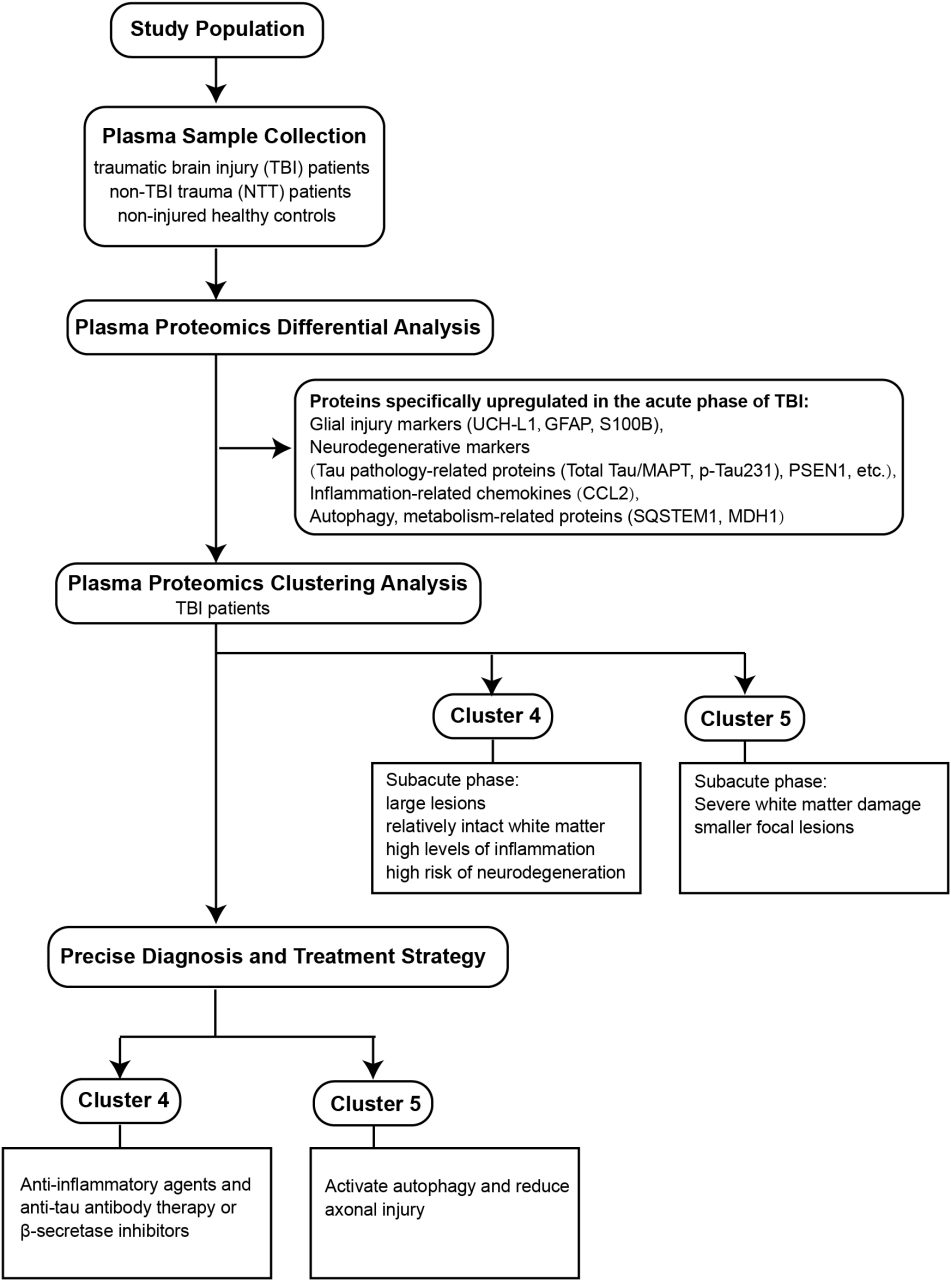
A flowchart revealing the molecular subtypes of traumatic brain injury patients through proteomics.

David J. Sharp’s team published an article titled “High-dimensional proteomic analysis for pathophysiological classification of traumatic brain injury.” The research draws its samples from the BIO-AX-TBI study, a comprehensive longitudinal cohort investigation of traumatic brain injury (**Figure 1**). By collecting a broad range of clinical and biological data, the research team created a unique opportunity to explore the intricate relationships between acute-phase protein expression dynamics and subsequent white matter injury and lesion progression. Breaking new ground in TBI clinical research, the study employed the Alamar NULISA™ central nervous system disease panel to comprehensively map plasma proteomic responses in acute TBI patients. By comparing these profiles with those of non-TBI trauma patients and healthy controls, researchers were able to identify previously unexplored protein signatures. Notably, the panel captured 120 distinct proteins, most of which had remained uncharacterized in prior human TBI investigations. To ensure methodological rigor, the team cross-validated their protein detection findings using OLINK^®^ proximity extension assay (PEA) and traditional ELISA techniques, thereby substantially increasing the reliability and reproducibility of their research conclusions.

This study utilized the Alamar NULISA™ CNS disease panel protein detection platform to identify 71 differentially expressed proteins in the plasma of traumatic brain injury (TBI) patients, with 16 exhibiting TBI-specific alterations. These proteins include neuroglial injury markers (UCH-L1, GFAP, S100B), tau pathology-related proteins (total tau/MAPT, pTau231), neurodegenerative regulatory factors (PSEN1), inflammatory chemokines (CCL2), and autophagy-metabolism pathway proteins (SQSTM1, MDH1). Coupled with diffusion tensor imaging (DTI) analysis, the findings indicate a significant reduction in white matter integrity during the subacute phase of TBI. Notably, elevated levels of CCL2 in the acute phase correlated with decreased fractional anisotropy (FA) values in the corpus callosum, supporting the hypothesis that CCL2 exacerbates white matter damage by promoting neuroinflammation. Additionally, increased levels of SQSTM1 were associated with elevated FA values across the entire skeleton, suggesting that autophagy activation may alleviate axonal injury by clearing damage-associated molecules. These findings provide direct evidence for the molecular mechanisms underlying white matter injury following TBI and imply that targeting inflammatory and autophagy pathways may represent potential strategies for improving TBI outcomes. Furthermore, a positive correlation between acute-phase focal lesion volume and the levels of UCH-L1, PSEN1, and pTau231 indicates that these proteins could serve as biomarkers for assessing injury severity. This study systematically reveals the molecular pathological features of TBI during the acute phase and their association with secondary neural injury, thereby offering new insights for protein-based precision diagnostic and therapeutic strategies in the management of TBI.

This study conducted a clustering analysis of acute-phase plasma proteins to categorize patients with TBI into five subgroups. Notably, clusters 3, 4, and 5 were primarily composed of TBI patients, significantly distinguishing them from the control groups represented by clusters 1 and 2. The clustering analysis revealed two distinct TBI subtypes: Cluster 5 exhibited the most severe subacute white matter damage, as indicated by the lowest z-scores for fractional anisotropy (FA) in both the entire skeleton and the corpus callosum, despite having relatively small focal lesion volumes. Conversely, Cluster 4 displayed the largest lesion volumes while maintaining relatively preserved white matter integrity, with higher FA z-scores. Importantly, the differences between these two clusters could not be explained by lesion dynamics or secondary white matter degeneration. Proteomics further demonstrated that inflammatory body-related proteins (IL-18), pro-inflammatory cytokines (IL-7), and neurodegenerative markers (SCNA, SOD1, TARDBP) were expressed at levels above the group mean in Cluster 4, whereas these proteins were significantly reduced in Cluster 5. Notably, despite the significant correlation of acute-phase molecular characteristics with neuroimaging injury patterns across the different subgroups, there were no statistically significant differences in the Glasgow Outcome Scale-Extended (GOS-E) scores at 6 and 12 months. This suggests that the acute-phase protein profile may not independently predict long-term functional outcomes, indicating the need for a comprehensive TBI prognosis assessment system that incorporates dynamic monitoring of multiple time-point biomarkers.

## Significance of the study

This research systematically reveals the molecular pathological heterogeneity of TBI and its clinical implications through a combined analysis of acute-phase plasma proteomics and neuroimaging. First, in the context of neurodegenerative risk prediction, the significant elevation of proteins such as pTau231, PSEN1, and total tau during the acute phase suggests that TBI may activate neurodegenerative pathways similar to Alzheimer’s disease through mechanisms involving tau hyperphosphorylation and amyloid precursor protein processing dysregulation. This finding supports the potential use of these proteins as early biomarkers to identify individuals at high risk for neurodegenerative changes post-TBI, thereby providing a critical window for long-term neuroprotective interventions, such as tau antibody therapies or β-secretase inhibitors.

Second, based on the pathological heterogeneity revealed through cluster analysis, we propose precision therapeutic strategies tailored to distinct TBI subgroups. Patients in Cluster 5 primarily exhibit significant white matter injury accompanied by smaller lesion volumes and lower inflammatory profiles; these individuals may benefit from interventions enhancing autophagy, such as rapamycin analogs, which could potentially mitigate axonal damage progression. In stark contrast, Cluster 4 is characterized by larger lesion volumes and elevated expression of proinflammatory proteins including IL18 and IL7; such patients may achieve superior clinical outcomes through anti-inflammatory therapies targeting the NLRP3 inflammasome. This molecular subtyping approach to individualized treatment offers a novel direction to overcome the persistent failure of clinical trials in TBI management.

Third, the translational potential of acute-phase protein monitoring was further validated in this study. The strong correlations between classical biomarkers such as NFL and GFAP with injury severity (e.g., positive correlation between UCH-L1 and lesion volume) support their utility as rapid triage tools for patient stratification in emergency settings. Concurrently, the bidirectional predictive functions of CCL2 and SQSTM1 on white matter injury provide molecular rationales for selecting individualized treatment strategies (such as inflammation modulation versus autophagy enhancement).

Finally, the interplay between neuroinflammation and degenerative pathology likely constitutes the core mechanism underlying long-term TBI sequelae. The co-expression patterns of proinflammatory factors (such as IL18) and neurodegenerative proteins (pTau231, SCNA) suggest that neuroinflammation may accelerate tau pathology and α-synuclein aggregation through microglial activation and blood-brain barrier disruption. This finding provides molecular evidence for the “second hit” hypothesis, wherein the acute inflammatory microenvironment potentially drives delayed neurodegeneration through epigenetic modifications or post-translational protein modifications. Future research should establish multi-timepoint dynamic monitoring models to delineate the critical nodes in the transition from acute protein profiles to chronic pathology, thereby optimizing intervention timing and target selection.

## Future directions

Traumatic Brain Injury (TBI) research is currently evolving from a descriptive phenotypic approach toward mechanism-driven precision interventions. Central to this transformation is the development of longitudinal monitoring systems that track patients across multiple timepoints, from acute injury through chronic phases. These systems integrate sophisticated liquid biopsies from plasma and cerebrospinal fluid with advanced neuroimaging modalities such as DTI and PET-tau/amyloid scanning. This comprehensive approach enables researchers to identify critical transition points—for instance, whether pTau231 peak concentrations drive subsequent tau pathological deposition—and threshold effects where specific protein concentrations might predict Alzheimer’s disease or CTE risk 5–10 years post-injury (8).

Recent cluster analyses have revealed distinct pathological subtypes among TBI patients, including pro-inflammatory (cluster 4) and autophagy-deficient (cluster 5) phenotypes. This heterogeneity suggests the need for tailored clinical trials: patients with predominantly inflammatory profiles might benefit from NLRP3 inhibitors like MCC950 or IL-1β antagonists, while those with autophagy deficiencies could respond better to rapamycin or lysosomal activators (9). Alongside these targeted approaches, developing dynamic biomarker profiles—such as CCL2/SQSTM1 ratios—could inform machine learning algorithms that guide real-time treatment decisions.

The future of TBI management lies in integrating dynamic biomarkers with spatial multi-omics and AI-driven prognostic models (8, 10–12). This multifaceted approach facilitates early risk stratification and subtype-specific interventions while proactively addressing potential neurodegenerative complications. By moving beyond the conventional one-size-fits-all approach toward precision medicine, we can meaningfully improve long-term neurological outcomes and quality of life for TBI patients.
